# miR-145 Alleviates Smooth Muscle Cell Phenotype Transition via ADAM17-Mediated ACE2 Shedding

**DOI:** 10.1155/2023/9497716

**Published:** 2023-07-20

**Authors:** Juan Wen, Baiyi Tang, Lan Guo, Wei Chen, Xiaohong Tang

**Affiliations:** ^1^Department of Cardiology, The Third Xiangya Hospital of Central South University, Changsha 410013, China; ^2^School of Medicine, Hunan Normal University, Changsha 410081, China

## Abstract

It has been shown that miR-145 is involved in the differentiation of vascular smooth muscle cells (VSMCs) and may regulate vascular remodeling. However, the molecular mechanisms behind these pathological processes in hypertension are not fully elucidated. The present study was to examine whether miR-145 modulates phenotypic transformation of VSMCs under normal state and synthetic state and to explore the possible role of ADAM17-mediated ACE2 shedding and ACE2-Ang-(1–7)-Mas receptor axis. Wistar rats were fed with high-sucrose/high-fat diet for 30 weeks to establish a metabolic hypertension animal model. VSMCs were cultured and treated with Ang II with or without miR-145 mimics or miR-145 inhibitor. Results showed the expression of contractile markers *α*-SMA and SM22*α*, miR-145, ACE2, and Mas receptor reduced in the thoracic aorta of metabolic hypertensive rats (MHRs), while that of synthetic marker OPN increased as compared to the control group. In in vitro study, miR-145 inhibitor inhibited the expression of *α*-SMA, SM22*α*, ACE2, Mas receptor, and the Ang-(1–7) excretion and induced the expression of synthetic markers OPN, EREG, and MMP2. However, miR-145 mimic produced opposite effects on the VSMCs. In addition, in the synthetic VSMC induced by Ang II, miR-145 inhibitor partially reversed the induced expression of OPN, EREG, and MMP2 by Ang II, while further decreasing the expression of *α*-SMA and SM22*α* and ACE2-Ang-(1–7)-Mas receptor. Cotreatment with ADAM17 siRNA partially reversed the inducible effect of miR-145 inhibitor on the EREG and MMP2, induced Ang-(1–7) excretion, and upregulated ACE2 and Mas receptor expression. In conclusion, miR-145 alleviates phenotype transition from contractile to synthetic type via ADAM17-mediated ACE2 shedding in VSMCs and retains the activation of ACE2-Ang-(1–7)-Mas axis, which may benefit the vascular structural remodeling in the metabolic hypertension.

## 1. Introduction

Hypertension is one of the most frequent cardiovascular disorders, and a leading global risk factor for death and disability worldwide [1]. It is reported that around 9.4 million deaths annually were attributed to hypertension [2], and the number of individuals diagnosed with hypertension is estimated to reach 1.5 billion globally by 2025 [1, 3]. Although treatment in hypertension can prevent the onset of cardiovascular events, existing therapies are only partially effective. The lack of definitive data as to the mechanisms for the hypertension contributes to this sign. A key pathological hallmark of hypertension is increased peripheral vascular resistance due to structural and functional changes in large (conductive) and small (resistance) arteries. Vascular remodeling is an important step in the occurrence and development of hypertension [4]. Smooth muscle cells (SMCs) are involved in the physiological and pathological vascular remodeling due to their remarkable ability to dynamically modulate their phenotypes. The phenotypic switching of SMCs refers to the shift between a differentiated “contractile” phenotype and a dedifferentiated “synthetic” phenotype in the SMCs. Synthetic phenotype is characterized by the loss of SMC markers, the increase in extracellular matrix (ECM) component synthesis, and the proliferation and migration of SMCs in response to various stimuli and environmental cues. Phenotypic evolution of SMC can be assessed by SMC markers, in vivo lineage-tracing and fate mapping [5, 6]. It has been established that *α*-SMA and SM22*α* are classical contractile markers and OPN, epiregulin (EREG), and matrix metalloproteinase-2 (MMP2) are synthetic markers [7, 8]. Adaptive phenotype alterations are essential for vascular development and repair, but dysregulation of this process plays a critical role in the pathological vascular remodeling [9]. The elucidation of mechanisms underlying the regulation of phenotype transition is of utmost importance for better understanding of vascular diseases.

MicroRNAs (miRNAs or miRs) are endogenous small noncoding RNAs that can regulate translation and degradation of messenger RNAs (mRNAs) at the post-transcriptional level [10–12]. miR-145 is a conserved miRNAs encoded by a bicistronic gene cluster and most frequently expressed miRNA in SMCs. It targets a network of transcription factors, including Kruppel-like factor 4 (Klf4), myocardin, and Elk-1 (ELK1, member of ETS oncogene family), to promote the differentiation and repress the proliferation of SMCs [13]. A disintegrin and metalloprotease 17 (ADAM17) is responsible for the EGFR transactivation, which subsequently modulates vascular remodeling. Compared with control mice, vascular ADAM17-deficient mice and ADAM17 antibody treated mice show alleviated cardiac hypertrophy, vascular medial hypertrophy, and perivascular fibrosis induced by Ang II via activating EGFR, which is independent of blood pressure regulation [14, 15]. ADAM17 governs angiotensin converting enzyme 2 (ACE2) shedding from cell membranes and promotes ACE/angiotensin II (Ang II) pressor and tissue remodeling action. It has been revealed that ADAM17 and miR-145 share the common binding domain, and ADAM17 is a direct substrate of miR-145 [16], which contributes to the migration and invasion of cancer [17–20]. However, the role of miR-145/ADAM17 pathway in the vascular remodeling in case of hypertension and the potential mechanisms is still poorly understood.

In the present study, the role of miR-145 pathway in the phenotype transition of VSMC induced by Ang II was investigated in vitro and in a rat model of metabolic hypertension, in which maladaptive vascular remodeling was obvious in our previous study [21]. Furthermore, the potential regulatory effects of miR-145/ADAM17 pathway on the vascular remodeling and RAAS activity were explored in vitro. Our findings will help reveal the role and mechanism of microRNAs in vascular remodeling of metabolic hypertension and provide experimental evidence for the development of new therapeutic targets for vascular remodeling.

## 2. Materials and Methods

### 2.1. Ethics Statement

This study was carried out with accordance to the recommendations in the Guide for the Care and Use of Laboratory Animals of the National Institutes of Health. The protocol was approved by the Animal Ethics Committee of the 3rd Xiangya Hospital of Central South University. All efforts were made to minimize suffering.

### 2.2. Materials

miR-145 mimics, miR-145 mimics negative control (NC), miR-145 inhibitor, and miR-145 inhibitor NC were designed and synthesized by Ribobio (Ribobio, China). Antibodies against angiotensin converting enzyme 2 (ACE2) (21115-1-AP), Mas receptor (20080-1-AP), osteopontin (OPN) (22952-1-AP), *α*-SMA (Proteintech, USA, 1 : 2000), SM22a (10493-1-AP), ADAM17 (20259-1-AP), EREG (CSB-PA189260), MMP2 (10373-2-AP) and GAPDH (10494-1-AP), and HRP goat anti-rabbit IgG (SA00001-2) were purchased from Proteintech (Proteintech, USA).

### 2.3. Metabolic Hypertension Model

The metabolic hypertension rat model was established as previously reported [21]. Male Wistar rats weighing 250–290 g were purchased from Beijing Vital River Laboratory Animal Technology Co Ltd. After 7-day acclimatization, male Wistar rats were randomly assigned to 2 groups. In the control group (Ctrl, *n* = 10), animals were fed with standard chow diet (animal experiment center of the Third Xiangya Hospital, Central South University, Changsha); in the metabolic hypertension group (MH, *n* = 12), animals were fed with a high-salt, high-fat diet (HSHF, standard diet 58%, lard stearin 12%, yolk power 10%, sugar 5%, peanut 5%, milk power 5%, salt 4%, and sesame oil 1%) together with 20% fructose in the drinking water for 30 weeks. The systolic blood pressure (SBP) was detected using a noninvasive computerized tail-cuff system (NIBP, Shanghai Alcott Biotech). Before the measurement, rats were warmed for 10–20 min at 28°C to detect the steady arterial pulsations of the tail artery. The SBP from 3 measurements was averaged for each animal. At the end of experiment, animals were euthanized with sodium pentobarbital (30 mg/kg, i.p), and the thoracic aorta was collected for histological examination as well as protein and RNA extraction.

### 2.4. Cell Culture

Rat arterial VSMCs (A7r5 cells) (ATCC, USA) were cultured in high-glucose Dulbecco's modified eagle medium (DMEM) (Hyclone, USA) containing 10% fetal bovine serum (FBS) (Gibco, USA) and 1% penicillin-streptomycin. A7r5 cells were plated in 6-well plates in DMEM without FBS. Twenty-four hours after passaging, the cells were treated with Ang II (1 *μ*M) with or without miR-145 mimics (100 nM) or miR-145 inhibitor (100 nM) as indicated in each experiment for 48 hours.

### 2.5. Construction and Transfection of Small-Interference RNA

On the basis of sequences of rat ADAM17, three kinds of custom stealth RNAi oligos (RiboBio Co. Ltd., China) were designed. The detailed sequence information and transfection efficiency are listed in Supplement Table 1 and Supplement Figure 1B. Specifically, the sequences used were 5′-GCATCATGTACCTGAACAA-3′ for ADAM17. A nonspecific control small-interference RNA (siRNA) (5′-CCAUGGCGCCAAUUCCAAACAGUUU-3′) was included in all siRNA experiments. All siRNA transfections were performed using Lipofectamine 2000 (Invitrogen) following the manufacturer's instructions. Briefly, A7r5 cells were seeded in a 96-well plate at a density of 3,000 cells/well in DMEM with 10% FBS and antibiotics. Twenty-four hours later, when cells confluence reached about 100%, the cells were transfected with siRNA (100 nM) using 5 *μ*l of Lipofectamine 2000 in 110 *μ*l of DMEM medium without FBS per well. Six hours after transfection, the medium was removed, and the cells were treated with various reagents for 48 h as indicated in each experiment. The proteins were extracted at the end of the experiment. The efficacy of ADAM17 silencing was verified by Western blotting.

### 2.6. Real-Time PCR

#### 2.6.1. RNA Extraction and Quantitative Real-Time Polymerase Chain Reaction (qRT-PCR)

Total RNA was extracted from tissues using TRIzol (Thermo Fisher Scientific, USA). cDNA was synthesized using a Transcriptor First Strand cDNA Synthesis Kit (CoWin Biosciences, China). qRT-PCR was performed using FastStart DNA Master SYBR Green (CoWin Biosciences, China) and amplification was performed with the Applied Biosystems 7500 Real-Time PCR system (Thermo Fisher Scientific, USA). Primer sets specific to miR-145, ADAM17, MASR, and *β*-actin were as follows: rat miR-145 primers were 5′-GCC TAC AGC CAT ACC ACC CGG AA-3′ (forward) and 5′-CCT ACA GCA CCC GGT ATC CCA-3′ (reverse), rat ADAM17 primers were 5′-GCG AGC TGA ACC TAA CCC AT-3′ (forward) and 5′-AAT CCT GCA TTG TCC CAC GAG-3′ (reverse), rat Mas receptor primers were 5′-CCT TTC AGT CCT CTA CCC CAT-3′ (forward) and 5′-ACT CTC TTC TCC GCT GTC A-3′ (reverse), rat *β*-actin primers were 5′-ACA TCC GTA AAG ACC TCT ATG CC-3′ (forward) and 5′- TAC TCC TGC TTG CTG ATC CAC-3′ (reverse). *β*-actin was used as an internal reference. The relative level was calculated using 2^−ΔΔCt^ method.

### 2.7. Western Blotting

Western blotting was performed as described previously [21]. Total proteins of tissues and cells were lysed in RIPA buffer (Beyotime, China). The concentration of proteins was determined with BCA (HonorGene, China) method. Equal amounts (40 *μ*g) of proteins were fractionated on 10% SDS-PAGE (Melonepharma, China) and transferred onto a nitrocellulose membrane (Millipore). The membrane was blocked with TBST buffer containing 5% nonfat milk for 2 hours at room temperature and incubated with antibodies against ACE2 (Proteintech, USA, 1 : 3000), Mas receptor (Proteintech, USA, 1 : 1500), OPN (Proteintech, USA, 1 : 3000), SM22 alpha (Proteintech, USA, 1 : 1000), *α*-SMA (Proteintech, USA, 1 : 2000), ADAM17 (Proteintech, USA, 1 : 500), *β*-actin (Proteintech, USA, 1 : 5000), or GAPDH (Proteintech, USA, 1 : 3000) overnight at 4°C. After washing in PBST 3 times, the membrane was incubated for 1.5 h at room temperature with horseradish peroxidase- (HRP-) conjugated secondary antibody (Proteintech, USA, 1 : 5000). The protein bands were visualized using a Typhoon scanner after treatment with super ECL Plus (Advansta, USA). *β*-actin and GAPDH were used as internal controls. The specific bands of target proteins were quantified with the Image Lab software.

### 2.8. Immunofluorescence Staining

Aortic tissue segments were harvested and stored at −80°C for immunofluorescence staining. Cryosections were incubated overnight at 4°C with primary antibodies against *α*-SMA (Proteintech, USA, 1 : 100), SM22*α* (Proteintech, USA, 1 : 50), and OPN (Proteintech, USA, 1 : 50). Biotin SP-conjugated AffiniPure donkey anti-rabbit IgG (Dianova GmbH, 1 : 300) secondary antibodies was added, followed by the addition of Cy3-conjugated streptavidin (Biotrend). The nuclei were stained with DAPI (Invitrogen in USA). The sections were mounted and observed with a Nikon confocal microscope; the fluorescence intensity was analysed using Nikon EZ-C1 3.90. The average values were calculated from 10 fields for 10 sections per rat.

### 2.9. ELISA

The supernatant of cells was collected and centrifuged at 1000 g at 4°C. Then, the supernatant was transferred to a sterilized tube. Ang-(1–7) ELISA kit (I190034618) was used to determine the level of Ang-(1–7) in the supernatant. All the procedures were carried out according to the manufacturer's instructions.

### 2.10. Luciferase Activity Assay

A 286bp sequence containing the predicted miR-145 binding site at the 3′-untranslated regions (UTRs) of ADAM17 or a 286bp sequence containing a scrambled sequence was cloned into the XhoI/NotI site of the psiCHECK-2 vector (HonorGene, China) to generate PsiCHECK-2-ADAM17-WT and PsiCHECK-2-ADAM17-mut vectors, respectively. For the luciferase assay, A7r5 cells were cultured in 12-well plates and cotransfected with 0.5 *μ*g of PsiCHECK-2-ADAM17-WT or PsiCHECK-2-ADAM17-mut and 50 nM of rno-miR-145-5p mimics or the negative control using the transfection reagent Lipofectamine 2000 (Invitrogen). 48 hours after transfection, the cells were harvested and luciferase assays were performed using the Dual-Luciferase Reporter Assay System (Promega) according to the manufacturer's instructions. Firefly luciferase was used for normalization. Experiments were repeated at least three times.

### 2.11. Statistical Analysis

All data are shown as mean ± standard error (SEM). One-way analysis of variance (ANOVA) following post hoc Student–Newman–Keuls test was used to determine the differences among multiple groups. A value of *P* < 0.05 was considered statistically significant.

## 3. Results

### 3.1. Vascular Remodeling and Phenotypes of VSMCs in Aortic Arteries of Hypertensive Rats

**Figure 1:**
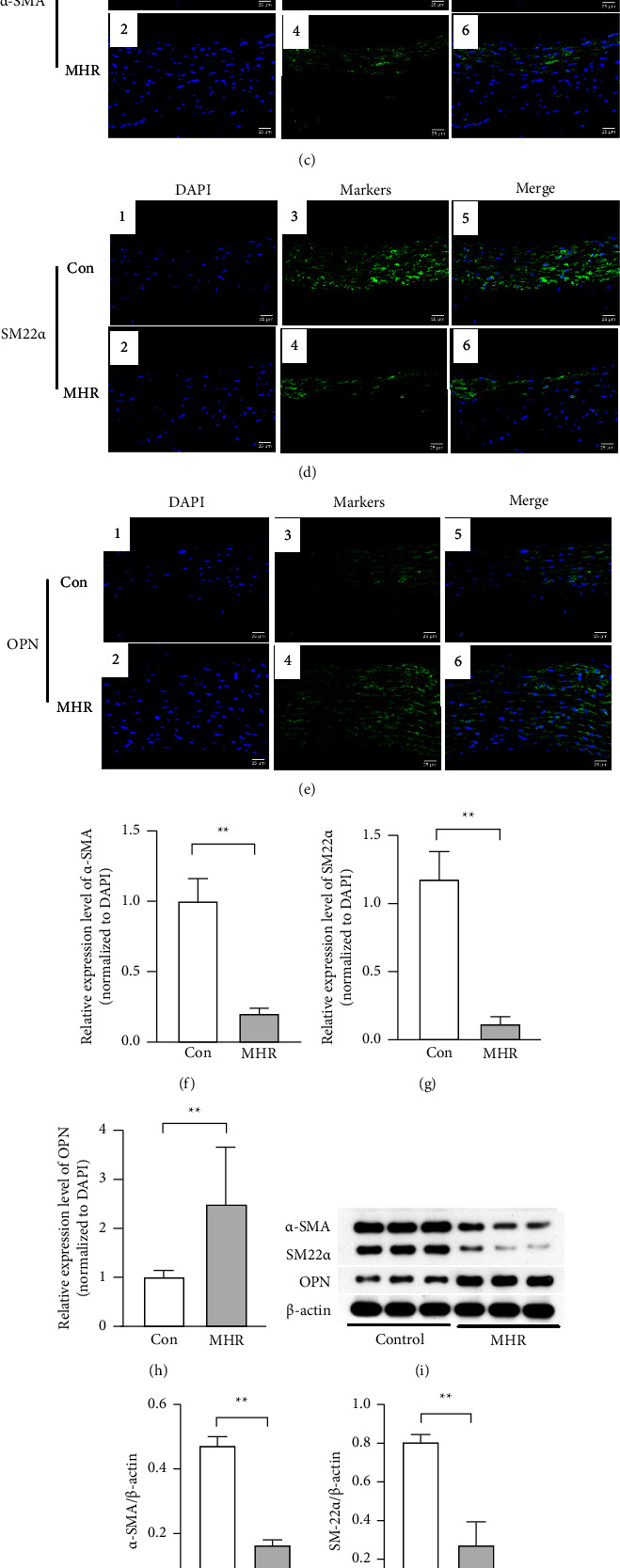
SMC phenotypes in aortic arteries of hypertensive rats. 22 male Wistar rats were randomly divided into control group (con group, *n* = 10) or metabolic hypertension group (MHR group, *n* = 12). Standard rat chow diet was administered to the control group and a high-sucrose/high-fat diet was administered to the MHR group. Immunoblot analysis: (c–e) the thoracic aorta was collected to detect *α*-SMA-, SM22*α*-, and OPN-positive cells by immunofluorescence staining (green), and nuclei were stained with DAPI (blue). (f–h) The quantification of *α*-SMA, SM22*α*, and OPN expression from panel (c–e). Western blotting: (j–l) The quantification of *α*-SMA, SM22*α*, and OPN expression in the aorta from panel (i) and the data were expressed after normalization to *β*-actin. (i) Representative figures from Western blotting of *α*-SMA, SM22*α*, OPN, and *β*-actin. (a, b) SBP and DBP were measured with the tail-cuff method every 6 weeks in groups. ^*∗∗*^*P* < 0.01 between groups, and ^*∗*^*P* < 0.05 between groups. ^#^*P* < 0.01 vs. baseline SBP. All the data are expressed as mean ± SEM. MHR, metabolic hypertension rats; SBP, systolic blood pressure; and DBP, diastolic blood pressure.

Rats were fed a high-sucrose/high-fat diet for 30 weeks to establish a rat MH model, which shows an obvious vascular remodeling [21]. Immunofluorescence staining and Western blotting were used to examine the location and expression of contractile markers (*α*-SMA and SM22*α*) and synthetic marker (OPN) in thoracic aorta, respectively. Compared to the control rats, MH rats showed a marked increase in the SBP after 6 weeks, which remained until 30 weeks (Figures 1(a) and 1(b)). *α*-SMA, SM22*α*, and OPN were mainly expressed in the cytoplasm of VSMCs (Figures 1(c)–1(e)). Western blotting and immunofluorescence staining showed that the expression of *α*-SMA and SM22*α* was significantly downregulated in the thoracic aorta of MH rats after 30 weeks, which was the most obvious in the VSMCs of the media layer (Figures 1(c),1(d), 1(f), 1(g), 1(j), and 1(k), *P* < 0.01). Interestingly, empty zone was observed in the medial wall of MHRs (Figures 1(c) and 1(d)). In addition, the expression of OPN significantly increased in the thoracic aorta of MH rats (Figures 1(e), 1(h), and 1(l), *P* < 0.01), which was observed in the VSMCs of medium and adventitia of aortic wall (Figure 1(e)).

### 3.2. Expression of miR-145, ADAM17, and ACE2-Ang-(1–7)-Mas Receptor Axis in Aortic Arteries of Hypertensive Rats

**Figure 2:**
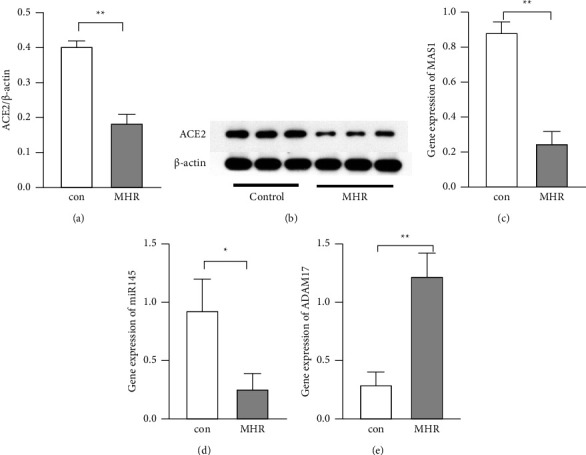
Expression of miR-145, ADAM17, ACE2, and Mas receptor in the aortic arteries of hypertensive rats. 22 male Wistar rats were selected randomly to receive standard diet or high-sucrose/high-fat diet for 30 weeks, the thoracic aorta was collected, the expression of ACE2 was detected by Western blotting, and the expression of MASR, miR-145, and ADAM17 was detected by qPCR. (a) Quantification of ACE2 expression from panel (b) data was expressed after normalization to the *β*-actin. (b) Representative figures from Western blotting of ACE2. (c–e) Quantification of mRNA expression of MASR, miR-145, and ADAM17 (qPCR). ^*∗∗*^*P* < 0.01 between groups and ^*∗*^*P* < 0.05 between groups.

To compare the expression of miR-145, ADAM17, ACE2, and MASR in the thoracic aorta between control rats and MH rats, Western blotting and real-time PCR were used to detect the protein and gene expression of these molecules, respectively. The miR-145 expression was significantly lower in the MH rats than in the control rats (Figure 2(d), *P* < 0.05). The mRNA expression of ACE2 and Mas receptor significantly reduced in the MH rats as compared to the control rats (Figures 2(a) and 2(c), *P* < 0.01). However, ADAM17 expression increased markedly in the MH rats (Figure 2(e), *P* < 0.01 vs control rats).

### 3.3. miR-145 Promoted VSMC Phenotype Transition from Synthetic to Contractile Phenotype and Augmented Activation of ACE2-Ang-(1–7)-Mas Receptor Axis

**Figure 3:**
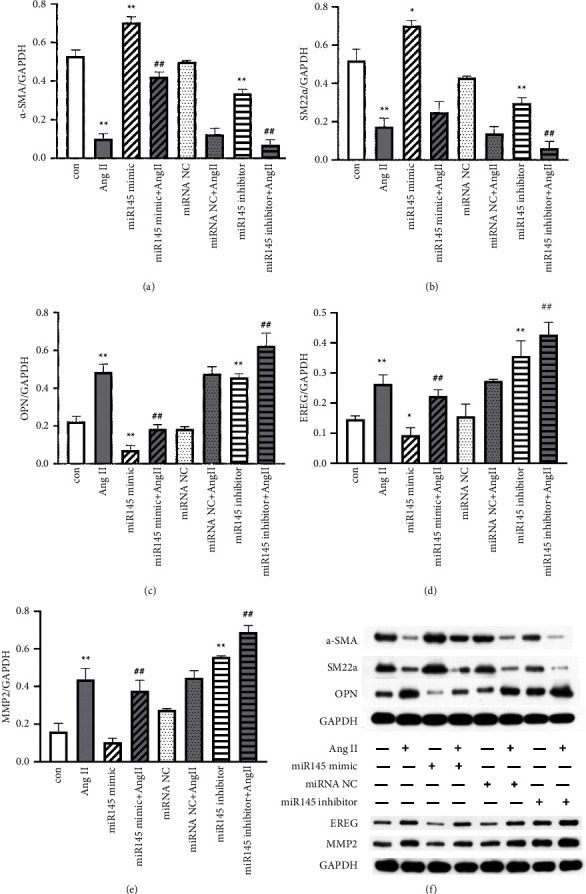
miR-145 modulates contractile and synthetic phenotype after Ang II treatment in vitro. VSMCs were treated with miR-145 mimic (100 nM) or miR-145 inhibitor (100 nM) in the presence or absence of Ang II (1 *μ*M) for 48 hours. (a–e) Protein expression of *α*-SMA, SM22*α*, OPN, EREG, and MMP2 from panel (f) and the data were normalized to that of GAPDH. ^*∗∗*^*P* < 0.01 vs. control group; ^*∗*^*P* < 0.05 vs. control group; ^##^*P* < 0.01 vs. Ang II group; and ^#^*P* < 0.05 vs. Ang II group. All the data are expressed as mean ± SEM from three independent experiments. miRNA NC, negative control miRNA.

To examine the effect of miR-145 on phenotype transition in VSMCs, Western blotting was used to detect the expression of synthetic and contractile markers. Results indicated that the gene expression of miR-145 was greatly promoted by miR-145 mimic and significantly inhibited by miR-145 inhibitor (*P* < 0.001, supplement figure 1C). The expression of *α*-SMA and SM22*α* in the miR-145 mimic-treated group increased significantly (Figures 3(a) and 3(b), *P* < 0.01), and the OPN and EREG expression decreased markedly as compared to the control group (Figure 3(c), *P* < 0.01). However, miR-145 inhibitor treatment significantly increased the expression of OPN, EREG, and MMP2, but decreased the expression of *α*-SMA and SM22*α* (Figures 3(a)–3(c), *P* < 0.01).

**Figure 4:**
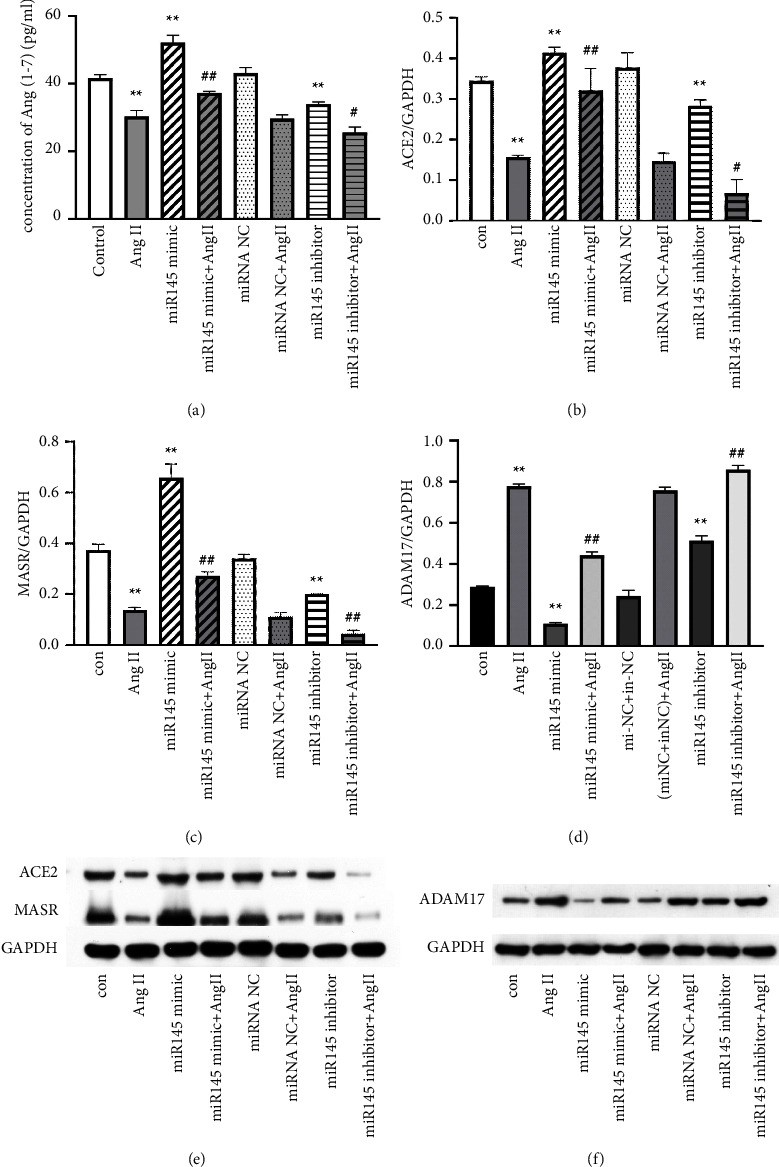
miR-145 augments Ang II-induced ACE2-Ang-(1–7)-Mas axis activation and ADAM17 expression in VSMCs. VSMCs were treated with control, Ang II (1 *μ*M), miR-145 mimic (100 nM), or miR-145 (100 nM) inhibitor alone or in combination for 48 hours. (a) Concentration of Ang-(1–7) in the supernatant (ELISA). (b, c) ACE2 and MASR expression from panel (e) and data are expressed as the fold of the GAPDH. (d) ADAM17 expression in groups from panel (f). (e, f) Representative figures of ACE2, MASR, and ADAM17 expression in VSMCs (Western blotting). ^*∗∗*^*P* < 0.01 vs. control group; ^*∗*^*P* < 0.05 vs. control group; ^##^*P* < 0.01 vs. Ang II group; and ^#^*P* < 0.05 vs. Ang II group. All the data are expressed as mean ± SEM from three independent experiments. miRNA NC, negative control miRNA.

To explore the effect of miRNA-145 on the activation of ACE2-Ang-(1–7)-Mas receptor axis, Western blotting was used to detect the expression of ACE2 and Mas receptor, and ELISA was applied to detect the concentration of Ang-(1–7). Our results showed that miR-145 mimics significantly increased the ACE2 (Figure 4(b)) and Mas receptor expression (Figure 4(c), *P* < 0.01) and induced Ang-(1–7) excretion in the cultured VSMCs (Figure 4(a), *P* < 0.01). Furthermore, miR-145 inhibitor treatment showed opposite results to miR-145 mimic on ACE2-Ang-(1–7)- Mas receptor axis (Figures 4(a)–4(c)).

### 3.4. miR-145 Modulated Ang II-Induced Phenotype Transition and ACE2-Ang-(1–7)-Mas Receptor Axis Activation

As Ang II is an effector molecule of the RAAS and can induce the phenotype transition of VSMCs from contractile phenotype to the synthetic phenotype, the effect of miR-145 on the phenotype of VSMCs induced by Ang II was further examined. Our results indicated that Ang II significantly enhanced the expression of synthetic marker OPN, EREG, and MMP2 (Figures 3(c)–3(e), *P* < 0.01), while markedly decreased the expression of contractile markers *α*-SMA and SM22*α* (Figure 3(a) and 3(b)) (*P* < 0.01). However, miR-145 mimic partially reversed the Ang II-induced reduction of *α*-SMA expression (Figure 3(a), *P* < 0.01) and mildly reversed Ang II-induced induction of OPN, EREG, and MMP2 expression (Figures 3(c)–3(e), *P* < 0.01). There was a reverse tendency of miR-145 mimic on the Ang II-induced reduction of SM22*α* expression (*P* > 0.05) (Figure 3(b)). Moreover, the specific miR-145 inhibitor amplified the effects of Ang II on SMCs. Interestingly, miR-145 mimic also partially reversed Ang II-induced reduction of ACE2 (Figure 4(b)), Mas receptor expression (Figure 4(c)), and Ang-(1–7) excretion (Figure 4(a)) (all *P* < 0.01), while miR-145 inhibitor further reduced the Ang II-induced inhibition on these markers (all *P* < 0.05).

### 3.5. Knockdown of ADAM17 Reversed Phenotype Transition Induced by miR-145* In Vitro*

**Figure 5:**
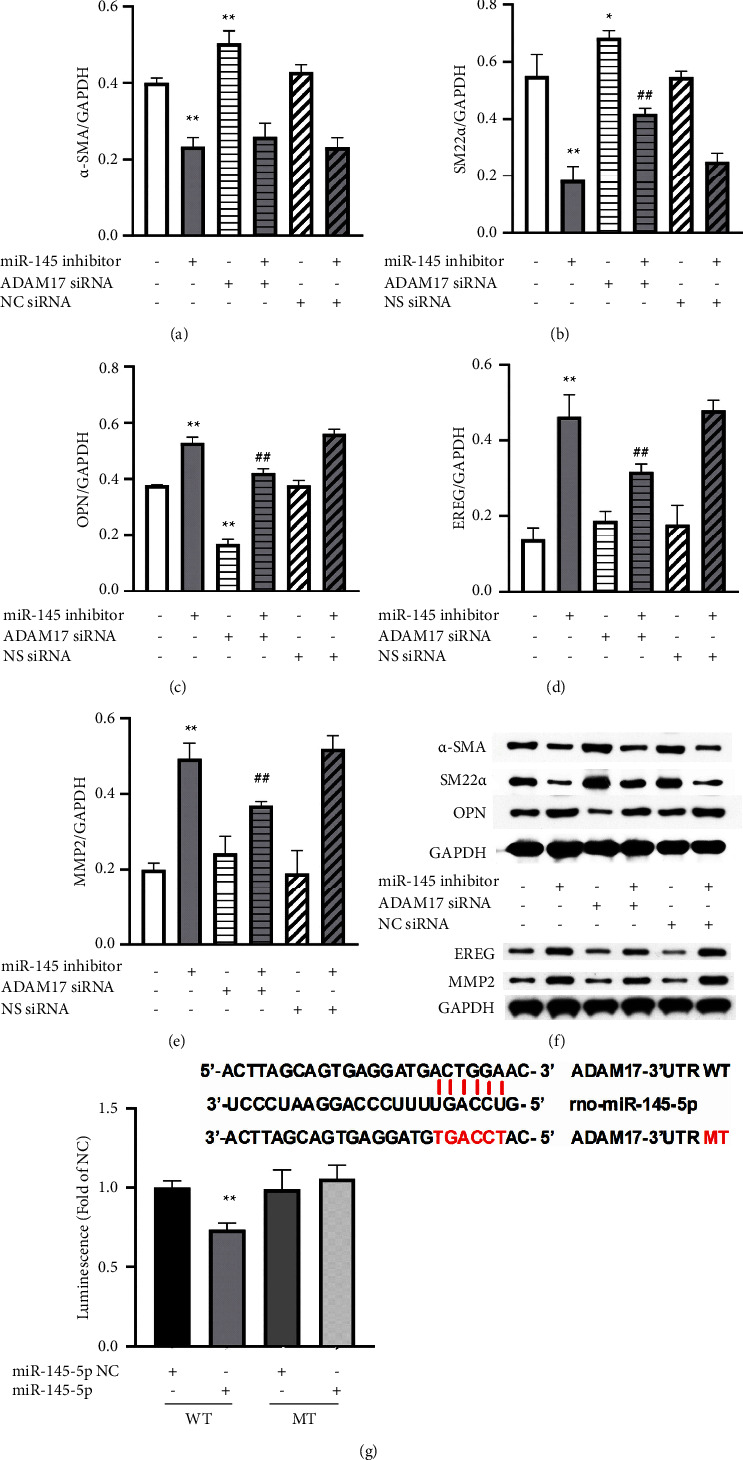
ADAM17 siRNA reversed phenotype transition induced by miR-145 in vitro. VSMCs were treated with miR-145 inhibitor in the presence or absence of ADAM17 siRNA (100 nM) as indicated for 48 hours. The expression of *α*-SMA (a), SM22*α* (b), OPN (c), EREG (d), and MMP2 (e) protein was detected by Western blotting. All the data were normalized to that of GAPDH. (f) Representative figures of OPN, *α*-SMA, SM22*α*, EREG, and MMP2 (Western blotting). (g) The luciferase reporter assay is shown. Cells were transfected with a reporter vector psiCHECK-2-ADAM17 3′-UTR plus either miR-145-5p or the negative control. ^*∗∗*^*P* < 0.01 vs. control group or miR-145-5p NC and ^##^*P* < 0.01 vs. miR-145 inhibitor group. All the data are expressed as mean ± SEM of three independent experiments. NC siRNA, negative control siRNA. WT, wide type. MT, mutant type.

ADAM17 is a direct substrate of miR-145, and whether ADAM17 mediates miRNA-145-induced effect on the phenotypic transition is still unknown. In our experiment, results showed miR-145 mimic inhibited ADAM17 expression and Ang II-induced ADAM17 expression (Figure 4(d), both *P* < 0.01), while miR-145 inhibitor further augmented Ang II-induced effect on ADAM17 (Figure 4(d), *P* < 0.01), which indicated that ADAM17 mediated the effect of miR-145 on the phenotype transition of VSMCs. Compared with the control group, ADAM17 knockdown promoted the expression of *α*-SMA (Figure 5(a), *P* < 0.01) and SM22*α* (Figure 5(b), *P* < 0.01) and inhibited the expression of OPN (Figure 5(c), *P* < 0.01). Of interest, ADAM17 itself had no effect on the expression of EREG and MMP2, while ADAM17 siRNA at least partially alleviated the induced effect of miR-145 inhibitor on these two synthetic markers (Figures 5(d) and 5(e), *P* < 0.01). To experimentally confirm that ADAM17 is a direct substrate of miR-145 by targeting the putative binding site, we performed luciferase reporters by cloning the wild-type 3′-UTR of ADAM17 or its mutant versions downstream of the firefly luciferase open reading frame. The result showed that miR-145 reduced the ADAM17 3′-UTR luciferase activity compared with the negative control, and no effects was observed on the mutated ADAM17 3′-UTR luciferase reporter (Figure 5(g)), indicating that ADAM17 is a direct target of miR-145. Therefore, miR-145 is involved in the phenotype transition likely via ADAM17 in the VSMCs.

### 3.6. ADAM17 Mediated miR-145-Induced Effect by Regulating ACE2-Ang-(1–7)-Mas Receptor Axis In Vitro

**Figure 6:**
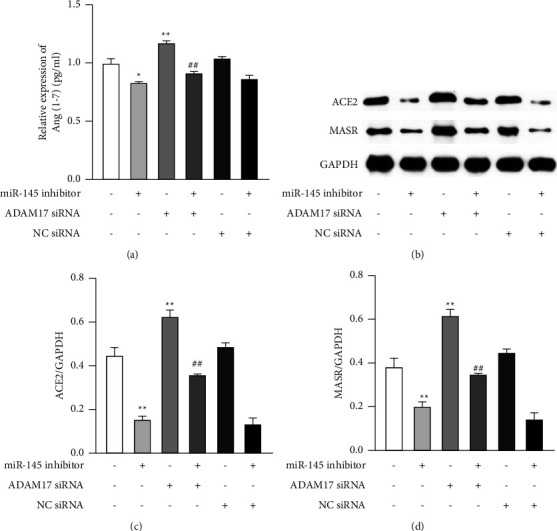
ADAM17 mediated miR-145-induced effect by regulating ACE2-Ang-(1–7)-Mas axis in vitro. VSMCs were treated with miR-145 inhibitor in the presence or absence of ADAM17 siRNA (100 nM) as indicated for 48 hours. (a) Concentration of Ang-(1–7) in the supernatant (ELISA). (b) Representative figures of ACE2 and MASR (Western blotting). Quantification of ACE2 (c) and MASR (d) expression determined by Western blotting, and the data were normalized to that of GAPDH. ^*∗∗*^*P* < 0.01 vs. control group; ^*∗*^*P* < 0.05 vs. control group; and ^##^*P* < 0.01 vs. miR-145 inhibitor group. All the data are expressed as mean ± SEM of three independent experiments. NC siRNA, negative control siRNA.

Regulation of ADAM17 could affect the effect of miR-145 on the phenotype transition in VSMCs, but the detailed mechanisms are largely unclear. We focused on the ACE2-Ang-(1–7)-Mas receptor axis, a typical pathway in the nonclassical renin-angiotensin-aldosterone system (RAS). siRNA was used to knockdown ADAM17 expression which induced Ang-(1–7) exection (Figure 6(a), *P* < 0.01), the expression of ACE2 (Figure 6(c), *P* < 0.01), and Mas receptor (Figure 6(d), *P* < 0.01). Treatment of VSMCs with ADAM17 siRNA also reversed the inhibitory effect of miR-145 inhibitor on the Ang-(1–7) level and the expression of ACE2 and Mas receptor (Figures 6(a)–6(d), *P* < 0.01). These indicate ADAM17 may modulate the effect of miR-145 on the phenotype transition through ACE2-Ang-(1–7)-Mas receptor axis in VSMCs.

## 4. Discussion

Our results revealed a novel role of miR-145 in regulating vascular remodeling. miR-145, which preserved the contractile phenotype of VSMC, was a robust inducer of ACE2-Ang-(1–7)-MAS axis, and ADAM17 modulated this effect.

Vascular remodeling is a characteristic pathological feature of hypertension, and VSMCs dysfunction is the important foundation of vascular remodeling. Phenotype transition of VSMCs is one key factor of vascular remodeling. miR-145 is a member of noncoding single stranded RNA, which has been proved to be highly expressed in VSMC and necessary for the differentiation and function of these cells, as well as an important determinant for phenotypic transition of VSMCs in response to vascular injury, such as subarachnoid hemorrhage [22]. In our study, the expression of miR-145 significantly decreased in rats with metabolic hypertension (Figure 2), which was consistent with findings from patients with essential hypertension [23]. miR-145 is able to regulate the proliferation and migration of VSMCs in spontaneous hypertension rats (SHRs) [24], while its function in phenotype transition of VSMCs is still unknown. Our findings for the first time indicated that miR-145 promoted the phenotype switch of VSMCs from synthetic to contractile phenotype (Figure 3). As Ang II is an important inducible factor of vascular remodeling in case of hypertension, Ang II was used to establish a cell model in which phenotype transition from contractile and synthetic phenotype was observed. These results showed that miR-145 attenuated Ang II-induced phenotype transition, which implies that miR-145 may be an important factor maintaining contractile phenotype in both normal VSMCs and pathological VSMCs.

ADAM17, as the most important sheddase, is also involved in the vascular remodeling in the case of hypertension. In our animal model, the ADAM17 expression significantly increased in the thoracic aorta of rats with metabolic hypertension as compared to the control group (Figure 2). There is evidence showing that vascular ADAM17-deficient mice and ADAM17 antibody treated mice have alleviated Ang II-induced cardiac hypertrophy, vascular medial hypertrophy, and perivascular fibrosis via activating EGFR, which is independent of blood pressure regulation [14, 25]. Our results further revealed that knockdown of ADAM17 with siRNA significantly increased the expression of contractile phenotype markers *α*-SMA and SM22*α* and decreased the expression of synthetic phenotype markers OPN (Figure 5), which indicate that ADAM17 is involved in the regulation of vascular remodeling.

Interestingly, in our experiment, miR-145 inhibited ADAM17 expression both in normal and pathological state (Figure 4(d)), which indicates that ADAM17 mediates the effect of miR-145 on the phenotype transition. Our study and other studies have revealed that ADAM17 and miR-145 share the common binding domain, and miR-145 can directly target the ADAM17 3′-UTR and suppress ADAM17 expression [26]. This ADAM17/EGFR/miR-145 feedback loop contributes to the migration and invasion of cancers such as nasopharyngeal cancer, colon cancer, hepatocellular cancer, renal cancer, and so on [16, 19, 27, 28]. However, the role of miR-145/ADAM17 feedback loop in the vascular remodeling in case of hypertension is poorly understood. In the present study, ADAM17 siRNA partially alleviated the induced effect of miR-145 inhibitor on the phenotype transition in VSMCs (Figure 5). Of interest, ADAM17 itself had no effect on the expression of EREG and MMP2, while cotreatment of ADAM17 siRNA reversed the induced effect of miR-145 inhibitor on these two synthetic markers, which indicates a regulatory role of ADAM17 in the miR-145-induced phenotypic switch. Thus, the miR-145/ADAM17 feedback loop may provide a new target for the treatment of vascular remodeling.

The potential downstream molecules involved in the miR-145/ADAM17 pathway were also explored in phenotype transition of VSMC. It has been confirmed that RAAS is involved in the regulation of vascular remodeling in case of hypertension [29], which involves two axes: ACE-Ang II-AT1 receptor axis and ACE2-Ang-(1–7)-Mas receptor axis. ACE2-Ang-(1–7)-Mas receptor axis is thought to be a nonclassical pathway and plays a protective role in the pathogenesis and development of hypertension [30, 31]. ACE2 cleaves the octapeptide Ang II to Ang-(1–7), a vasodilator peptide. This cleavage results in nitric oxide (NO) release and reduced sympathetic output, while restoring baroreflex sensitivity [32]. Similarly, our results showed that the expression of ACE2 and Mas receptor was significantly lower in the thoracic aorta of MH rats than in the control group (Figure 2). ADAM17, the most important sheddase, was also found to mediate the proteolysis and ectodomain shedding of ACE2 [33]. Our results suggest that knockdown of ADAM17 with siRNA can enhance the expression of ACE2 and Mas receptor and Ang-(1–7) concentration in both normal and pathological VSMCs. Moreover, miR-145 mimic increased the expression of these members (Figure 4), which supports the possible relationship between miR-145 and ACE2 in other published studies [34, 35]. In addition, ADAM17 siRNA reversed the effect of miR-145 on the ACE2-Ang-(1–7)-Mas receptor axis. Taken together, our findings suggest that ACE2-Ang-(1–7)-Mas receptor is a downstream pathway in the effect of miR-145/ADAM17 on the phenotype transition.

## 5. Conclusions

In conclusion, our study for the first time indicates that miRNA-145 induces aortic SMC phenotype transition via activating ADAM17, which may lead to vascular remodeling in the metabolic hypertension. Furthermore, ACE2-Ang-(1–7)-Mas receptor axis is involved in the regulation of miR-145/ADAM17 on the VSMCs. However, there were limitations in the present study. More studies are required to better understand the role of miR-145/ADAM17 in the pathophysiology of vascular remodeling in vivo and in the development of treatments for vascular remodeling due to hypertension.

## Data Availability

The data used to support the findings of this study are available from the corresponding author upon request.

## Supplementary Materials

Supplementary Materials The transfection efficiency of ADAM17 siRNA, miRNA-145 mimic, and miRNA-145 inhibitor on smooth muscle cells was list as supplementary data.
