# Supplementary material for: miR-145 Alleviates Smooth Muscle Cell Phenotype Transition via ADAM17-Mediated ACE2 Shedding
Source: Int J Hypertens. 2023 Jul 20;2023:9497716. doi: 10.1155/2023/9497716 (PMC10374374; doi:10.1155/2023/9497716)
Supplement: Supplementary Materials — The transfection efficiency of ADAM17 siRNA, miRNA-145 mimic, and miRNA-145 inhibitor on smooth muscle cells was list as supplementary data. [file 9497716.f1.doc]

**The transfection efficiency of ADAM17 siRNA, miRNA-145 mimic and miRNA-145 inhbitor on smooth muscle cells**

In order to test the transfection efficiency of ADAM17 siRNA, we designed three kinds of custom stealth RNAi oligos based on the sequences of rat ADAM17. The detailed sequence information lists in supplement table 1. A7r5 cells were treated with control and ADAM siRNAs(100nM), the protein was extracted at the end of experiment and the efficacy of ADAM17 silencing was verified by western blotting. The results shows that all three ADAM17 siRNA effectively sillence the protein level of ADAM17 in smooth muscle cells. Moreover, ADAM17 siRNA-1 was the most efficient than others (P<0.05, supplement figure 1B). So we chose ADAM17 siRNA-1 in experiments.

In order to test the efficiency of miR-145 mimic and inhibitor, A7r5 cells were treated with control, miRNA-145 mimic, non specific miRNA and miRNA-145 inhbitor. After treated for 48hours, cells were collected to test gene expression of miRNA-145 by Real time PCR (the detailed sequence information listed in the part of method). The gene expression of miR145 was greatly promoted by miR-145 mimic and significantly inhibited by miR145 inhibitor (P<0.001, supplement figure 1C).

Table 1 The sequence information of ADAM17 siRNA

| siRNAs | Sequence |
| --- | --- |
| ADAM17 siRNA-1 | 5’-GCATCATGTACCTGAACAA-3’ |
| ADAM17 siRNA-2 | 5’-CGAGTTGATAGCAAAGAGA-3’ |
| ADAM17 siRNA-3 | 5’-GGAGAAGAGAGCACTACTA-3’ |
| Non specific siRNA | 5’-CCAUGGCGCCAAUUCCAAACAGUUU-3’ |


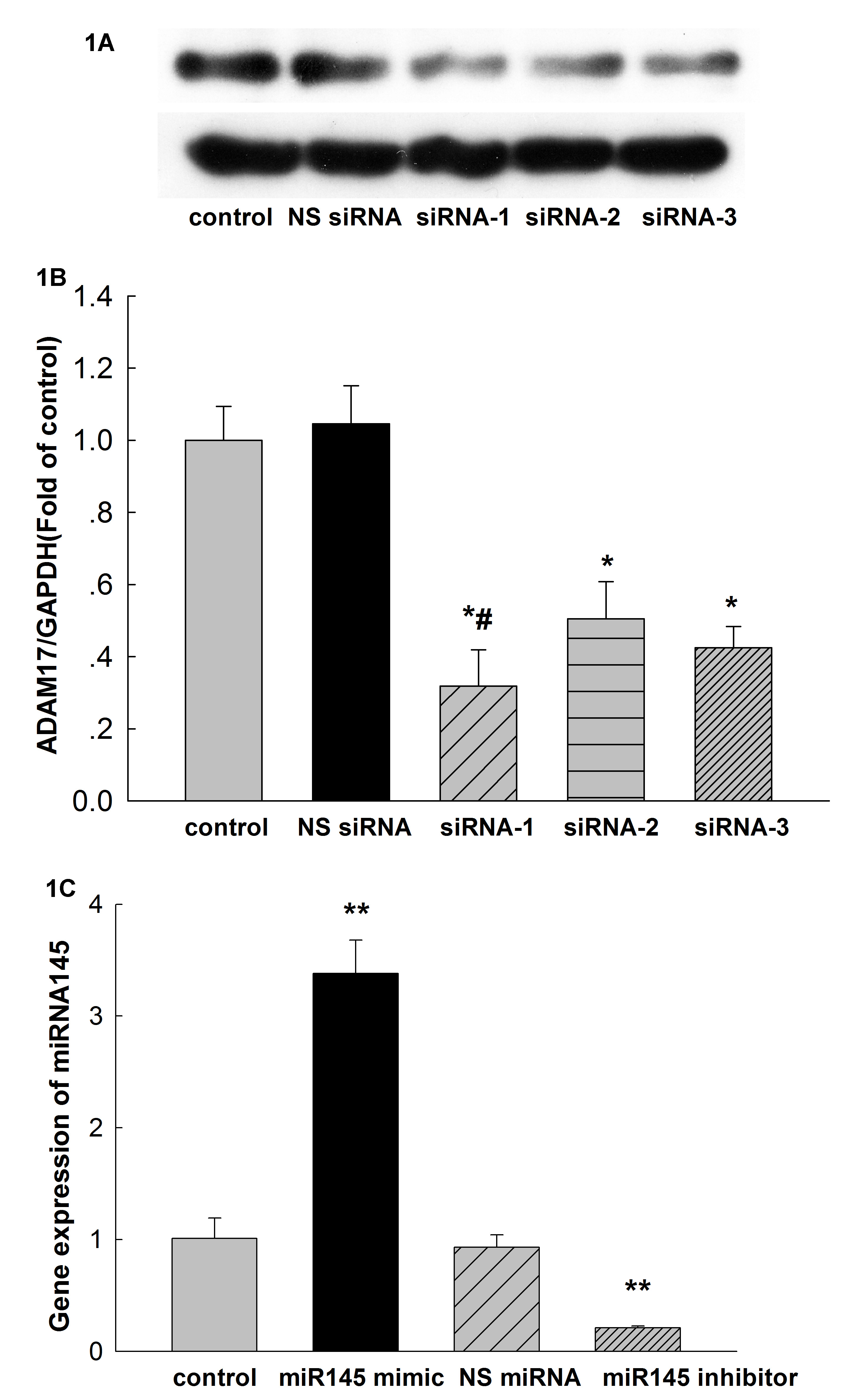


Supplement Figure 1. The transfection efficiency of ADAM17 siRNA, miRNA 145 mimic and miRNA 145 inhbitor.

VSMCs were treated with ADAM17 siRNA (100 nM) , miR145 mimic or miR145 inhibitor as indicated for 48 hours. The expression of ADAM17 (1B) was detected by Western blotting, the gene expressio of miR-145(1C) was detected by Real time PCR. All the data were normalized to that of GAPDH. (1A) representative figures of ADAM17 expression (Western blotting). **P<0.01 *vs.* control group; P<0.05 *vs.* control group; ^#^P<0.01 *vs.* ADAM17 siRNA-2 group. All the data are expressed as mean ± SEM of three independent experiments. NC siRNA,negative control siRNA.
